# Commentary: The unliganded glucocorticoid receptor positively regulates the tumor suppressor gene BRCA1 through GABP beta

**DOI:** 10.3389/fcimb.2015.00066

**Published:** 2015-09-24

**Authors:** Hanan Polansky, Adrian Javaherian

**Affiliations:** The Center for the Biology of Chronic DiseaseValley Cottage, NY, USA

**Keywords:** microcompetition, latent virus, transcription factor, cancer, stress

A significant event leading to the development of breast cancer is loss of BRCA1 function. BRCA1 is a tumor suppressor involved in the maintenance of genomic stability and prevention of cell transformation. Many studies showed that stress increases the binding of cortisol to the glucocorticoid receptor (GR) (Ritter et al., 2012). Ritter et al. showed that GR interacts with GABPβ at the BRCA1 promoter (Ritter et al., 2012). This, in turn, activates BRCA1 expression. The study also showed that addition of hydrocortisone, which binds the GR, eliminates the interaction between GR and GABPβ, causing a deficiency of the GABP transcription factor to the BRCA1 gene, which decreases its expression, and increases the risk of breast cancer (see Figure 1A). We would like to propose a second mechanism by which stress causes a deficiency of the GABP transcription factor involving the presence of certain latent viruses in the cell. This event has been described in 2003 in a book on Microcompetition (Polansky, 2003).

**Figure 1:**
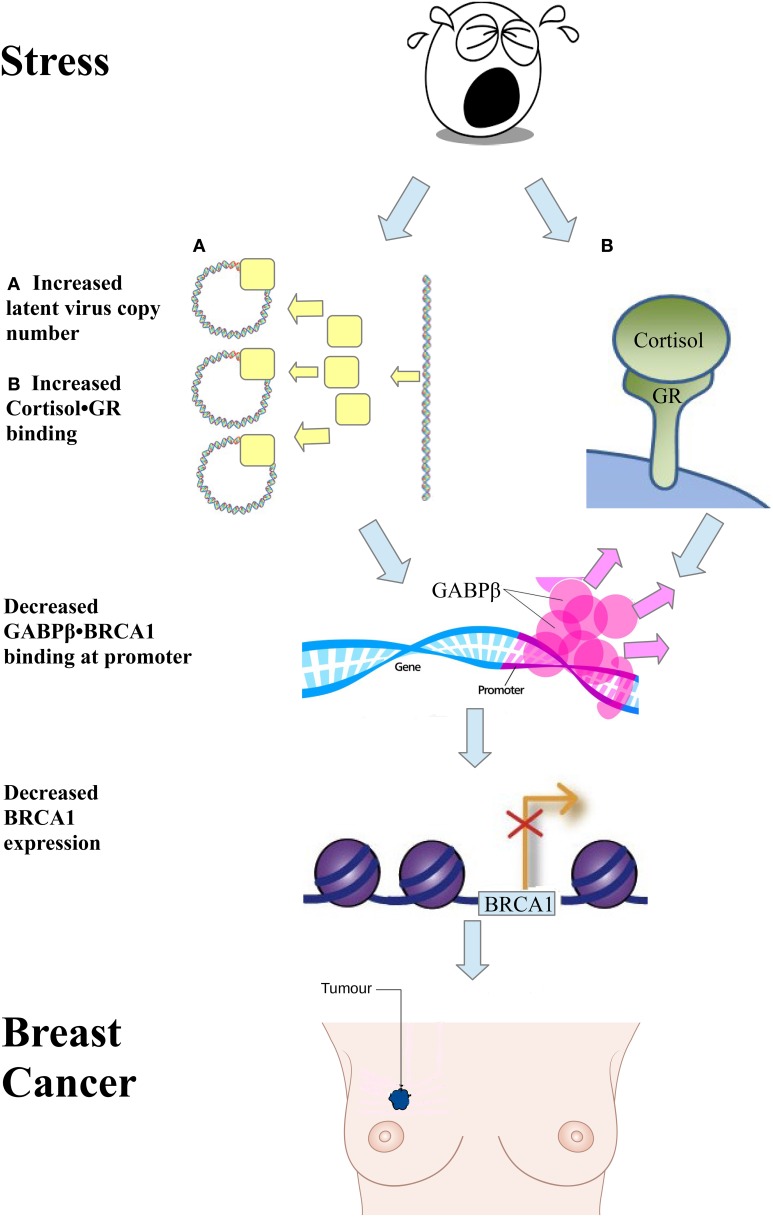
**Two pathways initiated by a stress signal lead to breast cancer**. One pathway involves an increase in latent viral copy number **(A)**. Another pathway involves an increase in cortisol∙GR binding **(B)**. Both events lead to a decrease in GABPβ binding to the BRCA1 promoter, decrease in BRCA1 expression, and breast cancer.

Many viruses consist of a core binding sequence as part of their enhancers, termed the N-box. When such a virus establishes a latent infection, the viral N-boxes bind the GABP·p300 transcription complex. Furthermore, since this complex is limiting, the viral N-boxes decrease the availability of the complex to cellular genes. As a result, the cellular genes that are stimulated by the GABP·p300 complex produce fewer proteins, and the genes that are suppressed by this complex produce more proteins. The abnormal levels of these cellular proteins induce disease (Polansky, 2003). The term “Microcompetition” describes the relationship between viral and cellular regulatory elements.

It is interesting that many common viruses, which establish a latent infection, have a strong N-box in their promoters/enhancers. These viruses include the Epstein-Barr virus (EBV), Cytomegalovirus (CMV), Herpes Simplex Virus (HSV), Varicella Zoster Virus (VZV), Hepatitis B Virus (HBV), Hepatitis C Virus (HCV), and the Human Papillomavirus (HPV). In fact, the CMV has the strongest promoter/enhancer known to science. In order to estimate the power of the CMV promoter, we will combine the results from a few studies. Results from Liu et al. (2004) can be used to estimate the strength of the CMV promoter/enhancer, which includes the N-box, relative to the strength of the promoter of the cellular platelet-derived growth factor-b chain (PDGF-b) gene. Part B of Figure 1 in Liu et al. reports the expression level driven by the CMV E/P (that is, the CMV enhancer/promoter) vs. those driven by the PDGF-b promoter in a variety of cells, including the COS-7, KB3-1, U251, PC12, and C17.2 cells. A close inspection of the numbers on the y-axis, which are depicted logarithmically, suggests that the CMV P/E is about 150-fold stronger than the PDGF-b promoter. We suspect that the reason for the difference is the much higher affinity that the CMV promoter/enhancer has to the GABP·p300 transcription complex relative to the PDGF-b promoter. Slobedman and Mocarski showed that during latency, an infected cell harbors about 10 copies of the CMV (Slobedman and Mocarski, 1999). Therefore, the impact of a latent infection with the CMV is equivalent to the introduction of 1500 copies of additional PDGF-b genes into the cell. Adam et al. showed that PDGF-b is susceptible to microcompetition with CMV (Adam et al., 1996). Therefore, according to Microcompetition theory, a latent CMV infection would result in a decrease in PDGF-b transcription followed by a decrease in the concentration of the expressed protein in the latently infected cell, ultimately leading to disease.

A number of studies showed the presence of EBV in breast cancer tissue. Mazouni et al. reported that 33.2% of tumors analyzed contained the *Bam*HIC sub-region of the EBV genome which encodes the Epstein-Barr encoded RNAs. Furthermore, EBV-positive tumors presented with a more aggressive phenotype (Mazouni et al., 2011). Studies have also shown a correlation between HPV and breast cancer. Damin et al. reported HPV DNA detected in 24.75% of breast carcinomas (Damin et al., 2004). El-Shinawi et al. reported that 78.6% of inflammatory breast cancer (IBC) tissues were HCMV-DNA positive, suggesting a possible role for HCMV in the pathobiology of IBC (El-Shinawi et al., 2013).

Stress increases viral copy number in a cell infected with a latent virus. This increase in viral copy number causes an increase in GABP bound to viral enhancers. Microcompetition ensues, decreasing the availability of GABP to the BRCA1 promoter, resulting in GABP transcription factor deficiency. This, in turn, decreases BRCA1 expression and increases the risk of breast cancer (see Figure 1B).

Due to the prevalence of latent viral infection, we believe that viral induced transcription factor deficiency is highly important, and has a significant impact on an individual's general health. Most individuals harbor a latent viral infection exposing them to transcription factor deficiency. Therefore, these individuals are at risk of diseases that can be triggered by such deficiency, including cancer.

## Conflict of interest statement

The authors declare that the research was conducted in the absence of any commercial or financial relationships that could be construed as a potential conflict of interest.
